# Analysis of the Li-Fraumeni Spectrum Based on an International Germline *TP53* Variant Data Set: An International Agency for Research on Cancer *TP53* Database Analysis

**DOI:** 10.1001/jamaoncol.2021.4398

**Published:** 2021-10-28

**Authors:** Christian P. Kratz, Claire Freycon, Kara N. Maxwell, Kim E. Nichols, Joshua D. Schiffman, D. Gareth Evans, Maria I. Achatz, Sharon A. Savage, Jeffrey N. Weitzel, Judy E. Garber, Pierre Hainaut, David Malkin

**Affiliations:** 1 Pediatric Hematology and Oncology, Hannover Medical School, Hannover, Germany; 2 Department of Pediatrics, Grenoble Alpes University Hospital, Grenoble, France; 3 Institute for Advanced Biosciences, Institut National de la Santé et de la Recherche Médicale 1209 Centre National de la Recherche Scientifique 5309 Universitè Grenoble Alpes, Grenoble, France; 4 Department of Medicine, Hematology-Oncology, Perelman School of Medicine, University of Pennsylvania, Philadelphia; 5 Department of Oncology, St Jude Children's Research Hospital, Memphis, Tennessee; 6 Division of Pediatric Hematology/Oncology, Departments of Pediatrics and Oncological Sciences, Huntsman Cancer Institute, Salt Lake City, Utah; 7 Division of Evolution and Genomic Sciences, University of Manchester, Manchester Academic Health Science Centre, Manchester, England; 8 Oncology Center, Hospital Sirio-Libanes, Sao Paulo, Brazil; 9 Clinical Genetics Branch, Division of Cancer Epidemiology and Genetics, National Cancer Institute, National Institutes of Health, Bethesda, Maryland; 10 Latin American School of Oncology, Sierra Madre, California; 11 Harvard Medical School, Boston, Massachusetts; 12 Department of Medical Oncology, Dana-Farber Cancer Institute, Boston, Massachusetts; 13 Division of Population Sciences, Dana-Farber Cancer Institute, Boston, Massachusetts; 14 Division of Hematology/Oncology, The Hospital for Sick Children, Department of Pediatrics, University of Toronto, Toronto, Ontario, Canada

## Abstract

**Importance:**

Li-Fraumeni syndrome is a cancer predisposition syndrome that is associated with a high, lifelong risk of a broad spectrum of cancers that is caused by pathogenic *TP53* germline variants. A definition that reflects the broad phenotypic spectrum that has evolved since the gene discovery is lacking, and mechanisms leading to phenotypic differences remain largely unknown.

**Objective:**

To define the phenotypic spectrum of Li-Fraumeni syndrome and conduct phenotype-genotype associations across the phenotypic spectrum.

**Design, Setting, and Participants:**

We analyzed and classified the germline variant data set of the International Agency for Research on Cancer *TP53* database that contains data on a cohort of 3034 persons from 1282 families reported in the scientific literature since 1990. We defined the term *Li-Fraumeni spectrum* to encompass (1) *phenotypic Li-Fraumeni syndrome*, defined by the absence of a pathogenic/likely pathogenic *TP53* variant in persons/families meeting clinical Li-Fraumeni syndrome criteria; (2) *Li-Fraumeni syndrome*, defined by the presence of a pathogenic/likely pathogenic *TP53* variant in persons/families meeting Li-Fraumeni syndrome testing criteria; (3) *attenuated Li-Fraumeni syndrome*, defined by the presence of a pathogenic/likely pathogenic *TP53* variant in a person/family with cancer who does not meet Li-Fraumeni syndrome testing criteria; and (4) *incidental Li-Fraumeni syndrome*, defined by the presence of a pathogenic/likely pathogenic *TP53* variant in a person/family without a history of cancer. Data analysis occurred from November 2020 to March 2021.

**Main Outcomes and Measures:**

Differences in variant distribution and cancer characteristics in patients with a germline *TP53* variant who met vs did not meet Li-Fraumeni syndrome testing criteria.

**Results:**

Tumor spectra showed significant differences, with more early adrenal (n = 166, 6.5% vs n = 0), brain (n = 360, 14.17% vs n = 57, 7.46%), connective tissue (n = 303, 11.92% vs n = 56, 7.33%), and bone tumors (n = 279, 10.98% vs n = 3, 0.39%) in patients who met Li-Fraumeni syndrome genetic testing criteria (n = 2139). Carriers who did not meet Li-Fraumeni syndrome genetic testing criteria (n = 678) had more breast (n = 292, 38.22% vs n = 700, 27.55%) and other cancers, 45% of them occurring after age 45 years. Hotspot variants were present in both groups. Several variants were exclusively found in patients with Li-Fraumeni syndrome, while others where exclusively found in patients with attenuated Li-Fraumeni syndrome. In patients who met Li-Fraumeni syndrome genetic testing criteria, most *TP53* variants were classified as pathogenic/likely pathogenic (1757 of 2139, 82.2%), whereas 40.4% (404 of 678) of *TP53* variants identified in patients who did not meet the Li-Fraumeni syndrome genetic testing criteria were classified as variants of uncertain significance, conflicting results, likely benign, benign, or unknown.

**Conclusions and Relevance:**

The findings of this cohort study suggest that this new classification, Li-Fraumeni spectrum, is a step toward understanding the factors that lead to phenotypic differences and may serve as a model for other cancer predisposition syndromes.

## Key Points

**Questions:**

What is the phenotypic spectrum associated with variants in *TP53*, the gene variant in persons with Li-Fraumeni syndrome, and what mechanisms underlie phenotypic differences?

**Findings:**

In this cohort study, the phenotypes within the classification *Li-Fraumeni spectrum* were defined, and data from 3034 persons from 1282 families with data available in the International Agency for Research on Cancer *TP53* Database were analyzed and classified to reveal meaningful differences in the *TP53* variant distribution between patients who met vs those who did not meet Li-Fraumeni syndrome testing criteria.

**Meaning:**

The study results suggest that this classification is a potential step toward understanding the factors that lead to phenotypic differences in the Li-Fraumeni spectrum and may serve as a model for the reclassification of other hereditary conditions with an increased cancer risk.

## Teaser

This cohort study examines the phenotypic spectrum of Li-Fraumeni syndrome and conducts phenotype-genotype associations across the phenotypic spectrum.

## Introduction

**Figure 1.**
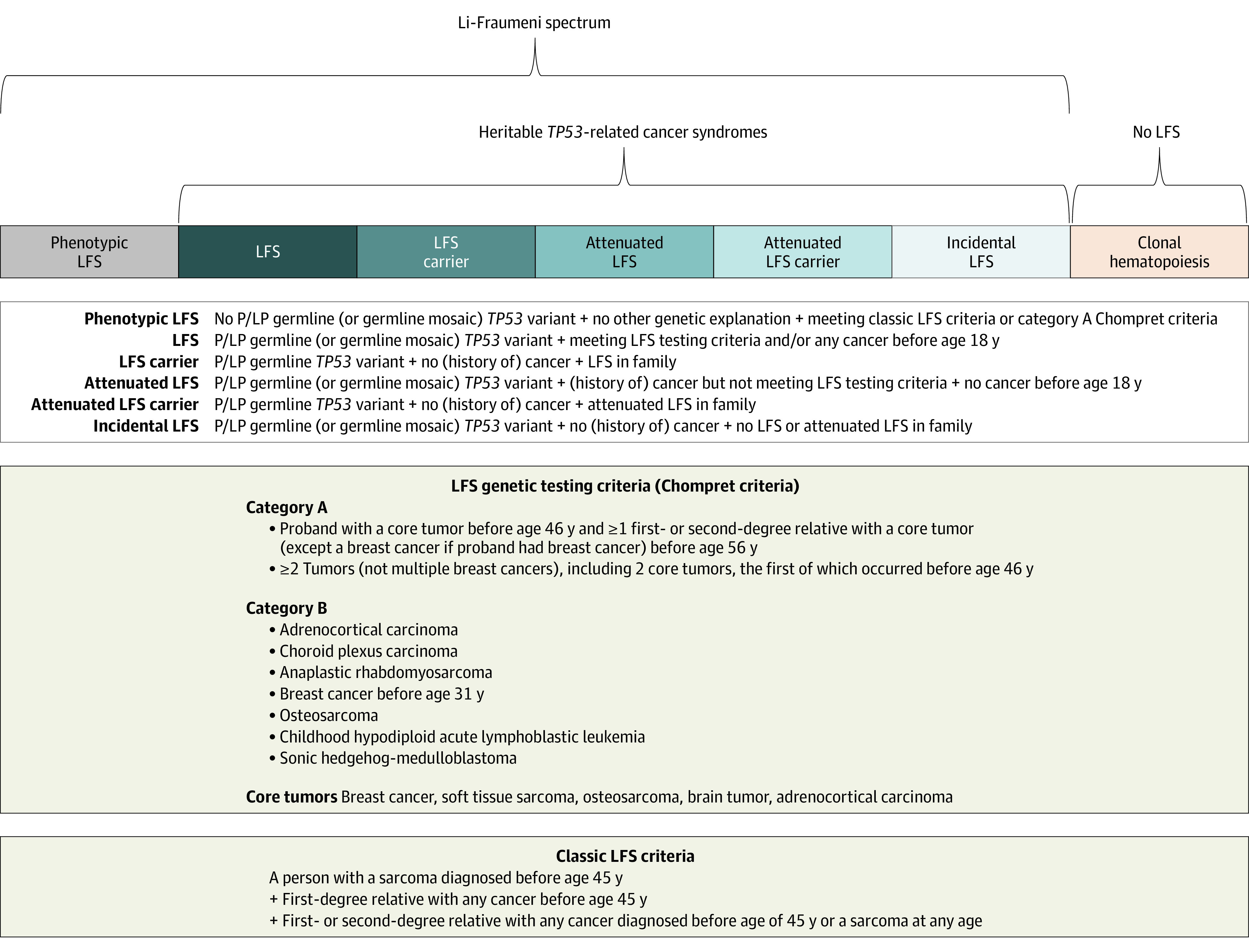
The Li-Fraumeni Spectrum and Heritable *TP53*-Related Cancer Syndromes For *TP53* variant classification we recommend *TP53*-specific guidelines.^7^ LFS indicates Li-Fraumeni syndrome; P/LP, pathogenic/likely pathogenic.

Li-Fraumeni syndrome (LFS; OMIM 151623) is a cancer predisposition syndrome that was first defined clinically by Frederick P. Li and Joseph F. Fraumeni Jr in 1969 based on their observation of a familial clustering of soft tissue sarcoma, breast cancer, and other neoplasms.^1,2,3,4^ In 1990, germline pathogenic variants of the *TP53* tumor suppressor gene were discovered and remain the only known cause of LFS.^5,6^ During the past 3 decades, LFS genetic testing criteria have evolved to address different scenarios regarding family and individual cancer histories, as well as specific tumor types that are associated with a germline *TP53* variant carrier state (Figure 1^7^).^8,9^

Cancers arising in individuals with LFS generally occur in age-related phases. They include adrenocortical carcinoma, choroid plexus carcinoma, rhabdomyosarcoma, and medulloblastoma that predominantly occur during the childhood phase (age 0-15 years); osteosarcoma, leukemia, and gliomas during the childhood-to-young adulthood transition phase; breast, gastrointestinal, and lung cancer and different sarcomas predominantly during the early adulthood phase (age 16-50 years); and pancreatic and prostate cancer that occur predominantly during the late adulthood phase (age 51-80 years).^10^

With the increasing use of gene panel–based analyses, *TP53* germline genetic testing is frequently performed in individuals who do not meet LFS genetic testing criteria,^11^ leading to the detection of *TP53* variant carriers, which are associated with a less penetrant phenotype.^11^ Germline *TP53* variants are also found through the increasing use of paired tumor/normal sequencing, and the American College of Medical Genetics and Genomics recommends reporting secondary and incidental *TP53* findings. A generally accepted definition that reflects the evolving phenotypic spectrum of LFS is lacking and, increasingly, individuals with a pathogenic/likely pathogenic (P/LP) *TP53* variant who do not meet the clinical LFS genetic testing criteria are being identified. In this article, we propose a definition that encompasses the phenotypic spectrum and have analyzed the distribution of *TP53* variants across this spectrum using data compiled in the *TP53* variant database from the International Agency for Research on Cancer (IARC) between 1994 and 2020 (release 20; http://p53.iarc.fr). Since 2021, this database is curated and maintained at the National Cancer Institute in Bethesda, Maryland.

## Methods

We aimed at defining the phenotypic spectrum associated with germline variants in *TP53* and to study genotype-phenotype associations across the spectrum. For this cohort study, we analyzed and classified the germline variant data set of the IARC *TP53* database that contains data on a cohort of 3034 persons from 1282 families reported in the scientific literature since 1990. The proposed definition of the *Li-Fraumeni spectrum* is depicted in Figure 1 and includes (1) *phenotypic LFS*, defined by the absence of a P/LP germline or germline mosaic *TP53* variant in persons who meets classic LFS criteria or category A Chompret criteria and (2) *LFS*, defined by the presence of a germline P/LP or mosaic *TP53* variant in a person with any cancer before age 18 years or who meets established testing criteria^1,2,3,4,8,9^ or additional cancer types associated with germline P/LP *TP53* variants, such as osteosarcoma,^12^ childhood hypodiploid acute lymphoblastic leukemia,^13^ and sonic hedgehog-medulloblastoma^14^ (additionally, through cascade genetic testing of blood relatives, cancer-free LFS carriers may be identified). It also includes (3) *attenuated LFS*, defined by the presence of a germline P/LP or mosaic *TP53* variant in a person with any cancer who does not meet LFS genetic testing criteria and has no cancer diagnosed before age 18 years (through cascade genetic testing of blood relatives, cancer-free attenuated LFS carriers may be identified); and (4) *incidental LFS*, defined by the presence of a germline P/LP or mosaic *TP53* variant in a person/family without cancer. Categories 2 through 4 within the Li-Fraumeni spectrum are termed *heritable TP53-related cancer syndromes* (Figure 1).^15^ Clonal hematopoiesis that is associated with somatic *TP53* variations can mimic a positive germline *TP53* genetic test result and is explicitly excluded from the Li-Fraumeni spectrum. The age threshold of 18 years was added to acknowledge the notion that any cancer occurring in infants, children, or adolescents with a P/LP variant in *TP53* does not represent an attenuated LFS phenotype. We propose the term *phenotypic LFS* only if a patient/family without a P/LP in *TP53* meets the classic LFS definition or selected Chompret subcriteria that describe the familial presentation or multiple tumors (category A; Figure 1). A patient without a P/LP variant in *TP53* who meets Chompret subcriteria that refer to specific cancer types (category B; Figure 1) is not deemed to have phenotypic LFS according to the proposed classification. In contrast, for patients with a P/LP in *TP53* who meet any of the Chompret criteria (categories A or B; Figure 1), the diagnosis of LFS is made.

The germline data set of the IARC *TP53* Database contains data on 3034 persons from 1282 families reported in the scientific literature since 1990. Among these 3034 persons, a total of 3305 cancers were diagnosed, including 361 patients with more than 1 cancer diagnosis. Patients included in this database were selected based on the underlying *TP53* variant and not on specific clinical criteria. The analysis of the germline data set is publicly available; thus, it does not require additional ethics review or informed consent.

To acknowledge the notion that the functional effects of many *TP53* variants are not entirely understood, we did not restrict this analysis to carriers of P/LP variants (according to http://ncbi.nlm.nih.gov/clinvar/). To reduce population-level bias, we excluded carriers of the p.R337H founder variant. This variant represents one of the most common variants in the IARC database because of the well-documented founder effect that is widespread in the Brazilian population (detected in up to 0.3% of the population of Southeastern Brazil). Studies in Brazilian carriers of this variant have shown variable patterns of individual and familial risk, ranging from fully penetrant LFS traits in a few families to asymptomatic carrier state in healthy participants who are detected by population-based screening. There is evidence that carriers of this variant are at high risk of childhood adrenocortical carcinoma and a high-to-moderate risk of several cancers that are typical of the LFS spectrum, including premenopausal breast cancer and soft tissue sarcoma. In most Brazilian carriers, the founder variant allele is embedded within an extended haplotype on chromosome 17p13, which may contain genetic modifiers. Recently, this region was found to harbor a variant in the tumor suppressor *XAF1* (E134*/Glu134Ter/rs146752602) in a subset of Brazilian p.R337H carriers. The compound variant haplotype was enriched in patients with cancer, conferring risk for sarcoma and subsequent cancers.^16,17^ The IARC germline *TP53* data set contains data on 282 Brazilian carriers of the p.R337H variant (8.4%). Of these carriers, 193 (68.4%) met LFS testing criteria, including 141 participants (50.0%) who met these criteria on the sole basis of diagnosis of adrenocortical carcinoma in the family. Twenty-eight patients (9.9%) did not meet LFS testing criteria, whereas 61 participants (21.6%) were carriers without cancer.

All statistical analyses were performed using Stata, version 14 (StataCorp). The Pearson χ^2^ test or Fisher exact test, when appropriate, was applied to compare proportions. A *P* value of <.01 was considered statistically significant.

## Results

Of the selected 3034 persons, 2139 (70.5%) fulfilled LFS genetic testing criteria or developed any cancer before age 18 years, 149 (4.9%) were unaffected carriers and blood relatives of these patients, 678 (22.3%) did not meet LFS genetic testing criteria and did not develop any cancer before age 18 years, and 33 (1.1%) were cancer-free carriers and relatives of these patients. Thirty-five variant carriers (1.2%) were identified incidentally. (Incidental LFS is defined in Figure 1: P/LP germline or germline mosaic *TP53* variant + no (history of) cancer + no LFS or attenuated LFS in the family). Whereas lifetime risk was equally high in patients who met vs did not meet LFS genetic testing criteria, tumor spectra showed significant differences (Table). Notably, there were more early adrenal, brain, connective tissue, and bone tumors in patients who met LFS genetic testing criteria, whereas carriers who did not meet LFS genetic testing criteria had a higher proportion of breast and other cancers, 45% of them occurring after age 45 years, which was consistent with the increasing use of comprehensive germline testing in patients with these tumor types. Because clinical LFS criteria are defined partly on age-related criteria, the 2 groups of individuals who met vs did not meet LFS clinical criteria showed a different age structure, with the age distribution of the group of patients not meeting clinical LFS criteria skewed toward older age. As a result, the median age of carriers who met LFS criteria was 26 years (range, 0-90 years), compared with 44 years (range, 18-91 years) in the group of patients who did not meet these criteria.

**Table.** Tumor Patterns in Patients Who Met vs Did Not Meet Li-Fraumeni Syndrome (LFS) Genetic Testing Criteria From the International Agency for Research on Cancer *TP53* Database (Release 20)

Characteristic	Genetic testing criteria, No. (%)^a^		*P* value
	Met^b^	Did not meet	
**Organ/tissue**			
Breast	700 (27.55)	292 (38.22)	<.001
Soft tissues	303 (11.92)	56 (7.33)	<.001
Adrenal gland	166 (6.53)	0	<.001
Brain	360 (14.17)	57 (7.46)	<.001
Bones	279 (10.98)	3 (0.39)	<.001
Hematopoietic/lymph nodes	129 (5.08)	43 (5.63)	.55
Lung	79 (3.11)	41 (5.37)	.003
Colon-rectum	81 (3.19)	36 (4.71)	.05
Ovary	30 (1.18)	24 (3.14)	<.001
Liver	27 (1.07)	4 (0.52)	.21
Prostate	33 (1.30)	10 (1.31)	.98
Skin	31 (1.22)	27 (3.53)	<.001
Stomach	77 (3.03)	24 (3.14)	.88
Kidney	11 (0.44)	11 (1.44)	.003
Pancreas	19 (0.75)	24 (3.14)	<.001
Not specified	136 (5.35)	59 (8.69)	.16
**Histologies, sarcoma, and brain tumors** ^c^			
Soft tissue sarcoma			
Fibrosarcoma	13 (4.3)	0	.23
Leiomyosarcoma	41 (13.5)	13 (23.2)	.06
Liposarcoma	18 (5.9)	9 (16.1)	.01
Rhabdomyosarcoma	116 (38.3)	6 (10.7)	<.001
Malignant fibrous histiocytoma	13 (4.3)	2 (3.6)	>.99
Other	26 (8.6)	4 (7.1)	>.99
Sarcoma not otherwise specified	76 (25.1)	22 (39.3)	.03
**Brain tumors**			
Astrocytoma	43 (11.9)	10 (17.5)	.24
Choroid plexus carcinoma	46 (12.8)	0	.002
Ependymoma	5 (1.4)	2 (3.5)	.25
Glioblastoma/glioma	45 (12.5)	23 (38.6)	<.001
Medulloblastoma	41 (11.4)	1 (1.8)	.02
Peripheral primitive neuroectodermal tumor	10 (2.8)	1 (1.8)	>.99
Other	17 (4.7)	6 (10.5)	.08
Cancer not otherwise specified	153 (42.5)	14 (24.6)	.01

^a^ Number and percentage of total number of cancers diagnosed in patients who met LFS genetic testing criteria: n = 2543 cases (in 2319 patients); and did not meet LFS genetic testing criteria: n = 762 cases (in 678 patients).

^b^ Including patients with any cancer before age 18 years.

^c^ Subset of cases for which detailed morphological information was available.

**Figure 2.**
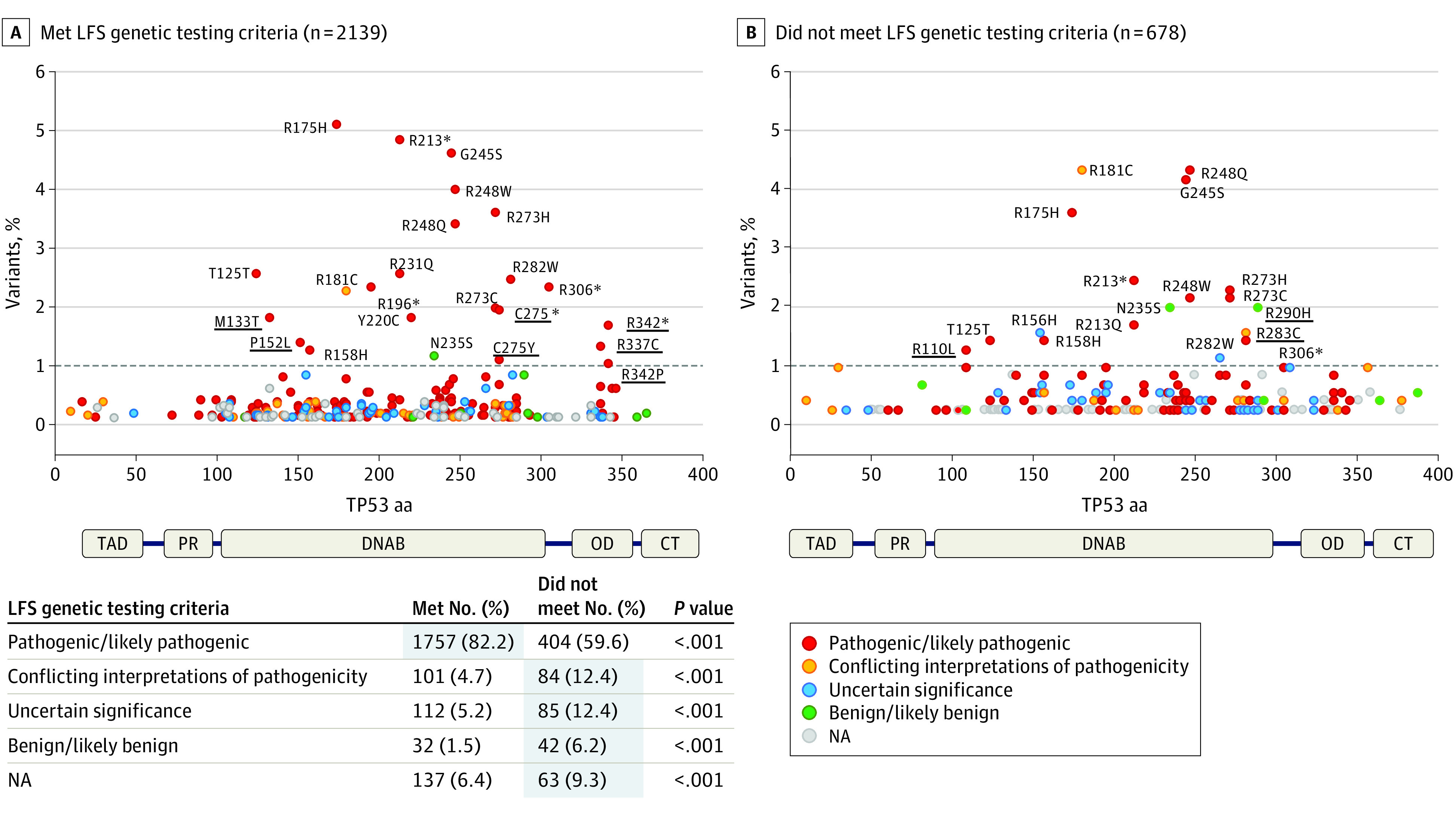
Variant Distribution in *TP53* Variant Carriers Who Met vs Did Not Meet Li-Fraumeni Syndrome (LFS) Genetic Testing Criteria A, Patients who met LFS genetic testing criteria and/or patients diagnosed before age 18 years. B, Patients who did not meet criteria. International Agency for Research on Cancer *TP53* database (release 20). Variants occurring in more than 1% of the data set are identified. Underlining indicates variants that differ between both data sets. aa Indicates amino acid; CT, terminal regulatory domain; DNAB, DNA-binding domain; NA, not applicable; OD, oligomerization domain; PR, proline rich region; TAD, transactivation domain.

Several hotspot variants (codons p.R175, p.G245, p.R248, p.R273, and p.R282) were present in both groups; however, specific P/LP variants were exclusively found in patients with LFS (p.M133T, p.P152L, p.C275Y, p.C275*, p.R337C, p.R342*, and p.R342P) while others were exclusively found in patients with attenuated LFS (p.R110L), a finding that requires validation. As expected, for patients who met LFS genetic testing criteria, most *TP53* variants were classified as P/LP (82.2%) (Figure 2, A), whereas 40.4% of *TP53* variants that were identified in patients who did not meet LFS genetic testing criteria were classified as variants of uncertain significance, conflicting results, likely benign, benign, or unknown (Figure 2, B). The group of patients who met LFS genetic testing criteria included 166 patients from families with childhood adrenocortical carcinoma, but no other cancers before age 18 years, 92 (55%) of whom had no reported family history of cancer. The variant spectrum in this subgroup was similar to patients who did not meet LFS criteria, with only 55% of the variants classified as P/LP.

## Discussion

We defined the phenotypes within the Li-Fraumeni spectrum and studied data from 3034 persons from 1282 families to reveal meaningful differences in the *TP53* variant distribution between individuals meeting vs those not meeting LFS testing criteria. The proposed classification has several advantages: it (1) reflects the spectrum of phenotypes associated with P/LP *TP53* variants; (2) describes the various phenotypes and does not rely on penetrance predictions; (3) uses established clinical criteria that were developed over decades^1,2,3,4,8,9^; (4) incorporates cancer risk–modifying factors without need to specify them (eg, a person with a hotspot variant may develop LFS while another person with the same variant may have attenuated LFS); and (5) has the potential to facilitate the search for risk modifiers in the future (eg, through genotype-phenotype associations or by studying risk modifiers in families with hotspot variants, such as p.R248Q, who developed LFS vs attenuated LFS). The disadvantages of the new classification are that: (1) probands may move between categories within the spectrum over time; (2) the classification has currently no immediate implications on cancer surveillance protocols because further risk analyses are required; and (3) carriers of *TP53* variants who do not fall into the P/LP variant category are not included in the new definition. While this separation is practical clinically, it may not reflect the true *TP53* biology, and variants of uncertain significance may be reannotated in the future.^7^

### Limitations

The limitations of our analysis are that: (1) we cannot rule out that a few patients with clonal hematopoiesis were misclassified as carriers of a germline *TP53* variant and entered into the germline data set of the IARC database^18^; (2) we were unable to provide data on the relevant group of patients with phenotypic LFS because only patients with a variant in *TP53* are included into the IARC database; and (3) population biases cannot be excluded.

## Conclusions

In this analysis, the tumor patterns observed in patients who did not meet clinical LFS criteria, such as breast, ovarian, skin, and pancreatic cancer, as well as histologies such as liposarcoma and glioblastoma, may primarily reflect the fact that carriers in this group develop cancers that are associated with the adult phase of LFS. Variants in this group may tend to be characterized by a reduced penetrance, leading to an older age of occurrence of cancers of the LFS spectrum. Further analyses of better characterized cohorts of patients with attenuated LFS and LFS are needed to confirm and further investigate the factors that determine whether a person with a P/LP *TP53* variant develops LFS or attenuated LFS. The proposed classification has several advantages for furthering clinical research in LFS and may serve as a model for the reclassification of other originally clinically defined genetic conditions. Patients classified as having LFS and attenuated LFS may need to be subdivided further in the future.
